# Irradiation Maintains Functional Components of Dry Hot Peppers (*Capsicum annuum* L.) under Ambient Storage

**DOI:** 10.3390/foods5030063

**Published:** 2016-09-12

**Authors:** Qumer Iqbal, Muhammad Amjad, Muhammad Rafique Asi, Aamir Nawaz, Samiya Mahmood Khan, Agustin Ariño, Tanveer Ahmad

**Affiliations:** 1 Institute of Horticultural Sciences, University of Agriculture, Faisalabad 38000, Pakistan; amjaduaf@gmail.com; 2 Nuclear Institute for Agriculture and Biology (NIAB), Faisalabad 38000, Pakistan; asimuhammad@yahoo.co.uk; 3 Faculty of Agricultural Sciences and Technology, Bahauddin Zakariya University, Multan 60000, Pakistan; aamirkhan_74@hotmail.com (A.N.); drsamiyakhan@hotmail.com (S.M.K.); 4 Instituto Agroalimentario de Aragón (IA2), Veterinary Faculty, Universidad de Zaragoza-CITA, 50013 Zaragoza, Spain; aarino@unizar.es; 5 Department of Horticulture, Faculty of Agricultural Sciences, Ghazi University, Dera Ghazi Khan 32200, Pakistan; horticulture.tanveer@gmail.com

**Keywords:** gamma radiation, hot pepper, capsaicinoids, antioxidants, quality, storage

## Abstract

Hot peppers used as natural flavoring and coloring agents are usually irradiated in prepacked form for decontamination. The effects of gamma radiation on the stability of functional components such as capsaicinoids and antioxidant compounds (carotenoids, ascorbic acid and total phenolics) were investigated in hot peppers (*Capsicum annuum*). Whole dried peppers packed in polyethylene bags were gamma irradiated at 0 (control), 2, 4, and 6 kGy and subsequently stored at 25 °C for 90 days. The irradiation dose did not substantially affect the initial contents of capsaicinoids, ascorbic acid and total phenolics, though the concentration of carotenoids declined by 8% from the control (76.9 mg/100 g) to 6 kGy radiation dose (70.7 mg/100 g). Similarly, during storage for 90 days at ambient temperature the concentrations of capsaicinoids and total phenolics remained fairly stable with mean percent reductions from 3.3% to 4.2%, while the levels of total carotenoids and ascorbic acid significantly (*p* < 0.05) declined by 12% and 14%, respectively. Overall, neither irradiation nor subsequent ambient storage could appreciably influence the contents of functional components in hot peppers. These results revealed that gamma irradiation up to 6 kGy can be safely used for decontamination to meet the needs for overseas markets without compromising product quality.

## 1. Introduction

Dry hot pepper (*Capsicum annum* L.) is one of the most important spices used worldwide as a natural flavoring and coloring agent owing to its unique spicy and pungent taste and color. In addition, peppers are a rich source of nutrients and diverse bioactive compounds with potential health-promoting properties, such as capsaicinoids and antioxidant compounds [1]. However, pepper is generally contaminated by molds, yeast and bacteria during the cultivation, drying, packaging, and storage processes. Therefore, to sanitize pepper, fumigation, steam heat sterilization, and irradiation are used to decontaminate undesirable microorganisms [2]. Interest in the ionizing radiation process is increasing because of persistently high food losses from infestation, contamination and spoilage, mounting concerns over food-borne diseases and growing international trade in food products that must meet strict import standards of quality and quarantine [3]. In Pakistan, a private-sector company initiated commercial food irradiation in 2010, and a total of 940 tons of legumes, spices, and fruits were processed in that year [4]. According to the Food Irradiation Treatment Facilities Database of the Joint FAO/IAEA Programme, around 50 tons of spices are irradiated per month in that Pakistani facility [5].

Ionizing radiation is effective against fungi common to hot peppers as well as total aerobic microorganisms. Previous work in our laboratory showed that total mold and Aspergillus counts in gamma-irradiated peppers achieved from 90% to 99% reduction at 2- and 4-kGy doses, respectively, while a radiation dose of 6 kGy eliminated the fungal population [6]. Song et al. [7] indicated that gamma irradiation at 5 kGy has potential for inactivating food borne pathogens (*Escherichia coli* O157:H7 and *Salmonella typhimurium*) in dried red pepper with minimal color changes. Similarly, Jung et al. [2] reported that a dose of 6 kGy reduced the population of total aerobic microorganisms effectively without affecting major quality indicators of red pepper powder, such as pungency and red color.

Postharvest gamma radiation up to 1 kGy did not significantly affect the quality of potatoes under non-refrigerated storage conditions [8]. Other studies carried out with pepper powder showed that radiation treatments have little impact on nutrients and overall quality under ideal storage temperatures [9,10]. However, the effect of irradiation treatments on the contents of bioactive compounds has not been thoroughly studied. Topuz and Ozdemir [11] recorded a significant decrease in carotenoids in sun-dried and dehydrated paprika subjected to irradiation. On the other hand, the concentration of capsaicinoids increased by 10% in irradiated paprika at 5 kGy [12]. What is largely unknown is the effect of subsequent storage and retail display under ambient conditions on functional compounds of irradiated whole dried peppers. Thus, the present study was designed to explore the impact of multilevel gamma-radiation doses (2, 4 and 6 kGy) and subsequent storage on capsaicinoids and antioxidant compounds in hot peppers.

## 2. Materials and Methods

### 2.1. Pepper Samples

Hot peppers (*Capsicum annuum* L.) were grown in plastic tunnels under drip irrigation system at the Vegetable Research Area of the Institute of Horticultural Sciences (University of Agriculture, Faisalabad, Pakistan) and harvested at the red ripe stage with pedicels. The fruits were dried in the sun for 6 to 8 days with an average daily temperature of 39 °C and relative humidity of 36% before the storage experiments. The moisture content of dried samples was determined with the air-forced oven drying method (indirect distillation at 105 °C), according to Method 44-15A of the American Association for Cereal Chemistry [13]. Moisture content in all hot pepper samples was 12%–13% after the sun-drying period.

### 2.2. Irradiation Treatments and Storage Conditions

Dried pods of hot pepper hybrids (200 g) were packed in synthetic low-density polyethylene bags (9 μm thick, 20 by 32 cm) and irradiated at dose levels of 0 (control nonirradiated samples), 2, 4, and 6 kGy in a Co60 gamma irradiator (Model Issledovatel, Mytishhi, Russia) using a dose rate of 0.4461 kGy/h at the Nuclear Institute for Food and Agriculture (NIFA), Peshawar, Pakistan. Upon irradiation, the samples were transported to the laboratory under refrigeration (4 °C), analyzed immediately (day 0), and stored at 25 °C for three months (day 90). Experiments were carried out in triplicate, and three subsamples of 25 grams were taken from each package and used for chemical analysis.

### 2.3. Determination of Capsaicin and Dihydrocapsaicin

A portion of 5 g from each subsample of hot pepper was oven-dried at 60 °C for 2–5 days, cooled and then ground to dried pepper powder. Samples were analyzed by using a chromatographic method previously described [14]. A mixture of 1-g sample with 10 mL acetonitrile was placed in 120-mL glass bottles with Teflon-lined lids, capped and placed in a water bath at 80 °C for 4 h and swirled manually every hour. The bottles were removed from the water bath and cooled at room temperature. The supernatant content of samples (2–3 mL) was filtered through a 0.45 μm filter (Millex^®^-HV filter) using a 5-mL disposable syringe (Millipore, Bedford, MA, USA) into an HPLC sample vial. For the liquid chromatographic analysis of capsaicinoids, an HPLC system LC-10 (Shimadzu, Kyoto, Japan) equipped with an SPD-10A. UV-Vis detector (set at 280 nm wavelength) was used. The analysis was carried out with the isocratic mobile phase (acetonitrile:water, 60:40) at a flow rate of 1 mL/min using a column Discovery C18 (250 × 4.6 mm, 5 μm) supplied by Supelco (Bellefonte, PA, USA). The limit of detection (LOD) was 0.1 μg/g for both capsaicinoids.

### 2.4. Total Carotenoids

Based on the Association of Official Analytical Chemists official Method 970.64 [15], two grams of hot pepper sample were ground using a mortar and pestle and transferred to a 100-mL flask covered with a stopper. The sample was blended for one minute with a mixture of 30 mL hexane:acetone:ethanol:toluene (10:7:6:7). For hot saponification, 2 mL of 40% methanolic KOH were pipetted into the flask, swirled for one minute and placed in a 56 °C water bath for 20 min. The sample was cooled for one hour in the dark, and then, 30 mL of hexane were pipetted into the flask, dried over anhydrous sodium sulfate made up to volume and shaken vigorously for one minute. Upper phase was 50 mL. Absorbance was measured at 436 nm using the IRMECO UV-Vis spectrophotometer Model U2020 with β-carotene (Sigma-Aldrich, St. Louis, MO, USA) as the standard.

### 2.5. Ascorbic Acid

Ascorbic acid was quantitatively determined according to the 2,6-dichlorophenolindophenol AOAC official Method 967.21 [15]. A sample of hot peppers (10 g) was blended with 2.5 mL of 20% metaphosphoric acid, and distilled water was then added up to the 100-mL mark. Ten milliliters of the suspension were titrated with freshly-prepared standard of 2,6-dichlorophenolindophenol dye until a light, but distinct rose pink color persisted for 15 s.

### 2.6. Total Phenolic Compounds

Total phenolic contents of hot peppers were analyzed using the modified Folin-Ciocalteu reagent method as described elsewhere [14]. About 0.5 g of the sample was macerated in 3 mL 80% aqueous acetone with a mortar and pestle. The extracts were placed into tightly-sealed micro-tubes and maintained in darkness at 4 °C overnight. Samples were centrifuged at 1000 rpm for 2 min. A mixture of 135 μL H_2_O, 750 μL 1/10 dilution Folin-Ciocalteu reagent (Sigma-Aldrich, St. Louis, MO, USA) and 600 μL 7.5% (w/v) Na_2_CO_3_ was added to 50 μL of extract in 1.5 mL micro-tubes. After vortexing for 10 s, the mixture was incubated at 45 °C in a water bath for 15 min. Samples were allowed to cool to room temperature before reading the absorbance at 765 nm using the IRMECO UV-Vis spectrophotometer Model U2020. A blank was prepared from 50 μL 80% aqueous acetone. The gallic acid standard curve was prepared from freshly-made 1 mg/mL gallic acid (Acros Organics, Geel, Belgium) in 80% aqueous acetone.

### 2.7. Statistical Analysis

Analysis of variance was computed with the data from each attribute using the STATISTICA Computer Program (Version 2003, StatSoft Inc., Tulsa, OK, USA). Experiments were performed according to a completely randomized design with factorial arrangement with three replicates for each treatment. The factor radiation dose had four levels (0, 2, 4 and 6 kGy) and the factor storage had two levels (0 and 90 days). There were eight treatment combinations and each treatment replicated thrice. The least significant difference test at the 5% level of probability was used to check the differences among mean values according to Hill and Lewicky [16].

## 3. Results and Discussion

The analysis of variance for the functional components of hot peppers is shown in Table 1. The analysis was conducted to determine whether there was significant difference between capsaicinoids and antioxidants with radiation treatments and storage time. Results indicated that radiation treatments had no significant effect on the concentrations of all chemical parameters under study. Storage duration, however, affected the levels of carotenoids and ascorbic acid significantly (*p* < 0.05); storage for 90 days resulted in significantly lower concentrations as compared to the day 0.

### 3.1. Capsaicin and Dihydrocapsaicin Contents

**Figure 1:**
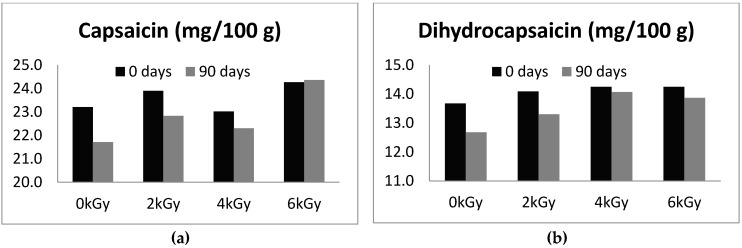
Effect of gamma radiation on (**a**) capsaicin and (**b**) dihydrocapsaicin concentrations in hot peppers during three months’ storage.

Hot pepper cultivars are rich in capsaicinoids, responsible for the specific taste of pepper fruits, which may also be used in pain relievers, due to their pharmacological properties [17]. On day 0 the concentration of capsaicin in control group (non-irradiated) was 23.2 ± 5.0 mg/100 g, and barely changed in 2 kGy (23.9 ± 5.5), 4 kGy (23.0 ± 5.6), and 6 kGy (24.3 ± 5.7) radiation dose treatment. Likewise, the levels of dihydrocapsaicin remained fairly stable from 13.7 ± 3.2 mg/100 g in control group to 14.1 ± 3.1 (2 kGy), 14.3 ± 3.1 (4 kGy), and 14.3 ± 2.7 (6 kGy) in irradiated samples. The effect of the subsequent storage during 90 days is presented in Figure 1. The levels of capsaicin and dihydrocapsaicin were essentially maintained during storage showing only minor losses of 3.4% and 4.2% on average, respectively. For both capsaicin and dihydrocapsaicin the lower losses during storage were observed in irradiated samples at 6 kGy.

The maximum concentration of capsaicin at day 0 was detected in the samples irradiated at 6 kGy (24.3 mg/100 g), and so it was at the end of the storage period at day 90 (24.4 mg/100 g). Our finding is correlated with Subbulakshmi et al. [18] that the pungency of irradiated paprika tended to be greater when compared with non-radiated control. Similarly, Topuz and Ozdemir [12] found that an increase in capsaicinoids with the effect of irradiation could be explained by changing the conformation of the molecules in the food matrix which affects the extraction yield. Doses up to 5 kGy of gamma irradiation led to capsaicinoids increases in paprika up to 10% in a dose-dependent manner.

### 3.2. Total Carotenoids

**Figure 2:**
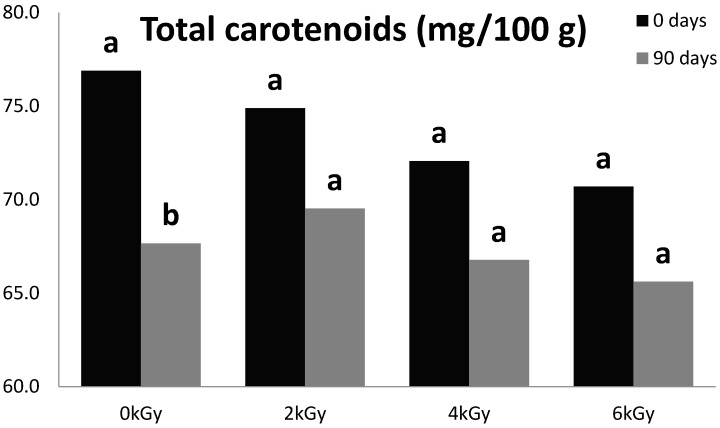
Effect of gamma radiation on total carotenoids concentration in hot peppers during three months’ storage; **a**, **b** indicate differences between these pairs of treatment means at *p* < 0.05.

Capsicum fruits have been used as natural food colorants, and an increasing interest is being paid to the spice red pepper because of its economic importance and diversified composition [19]. An immediate decrease of carotenoids was observed for all the radiation treatments as compared to the control, and further reductions were observed over time during 3 months of storage. The initial concentration of carotenoids in control group at day 0 was 76.9 ± 14.6 mg/100 g and levels gradually declined by 8% following treatment at radiation doses of 2 kGy (74.9 ± 13.9), 4 kGy (72.1 ± 13.1), and 6 kGy (70.7 ± 12.5). These results indicated that carotenoids are somewhat sensitive to gamma radiation as their concentrations decreased with increasing irradiation doses. This decreasing trend may be attributed to absorbed energy assisted by irradiation doses up to 6 kGy and/or increase in the rate of the oxidation reaction. It is well established that as ionizing radiation passes through food, it creates a trail of chemical transformations by primary and secondary radiolysis effects [20]. As shown in Figure 2, the evolution of carotenoids during storage showed significant losses of 12.0% in control peppers (*p* < 0.05).

Topuz and Ozdemir [11] observed in paprika that carotenoid reduction due to irradiation was possibly caused by an increase in oxidation reaction under gamma radiation and also secondary oxidative effects of free radical (H_2_O_2_, O_3_ and OH) formation during radiation. Significant losses of carotenoids (about 40%–60%) have been reported for cinnamon, oregano, parsley, rosemary, bird pepper, and sage after gamma irradiation at a dose of 10 kGy [21].

### 3.3. Ascorbic Acid

**Figure 3:**
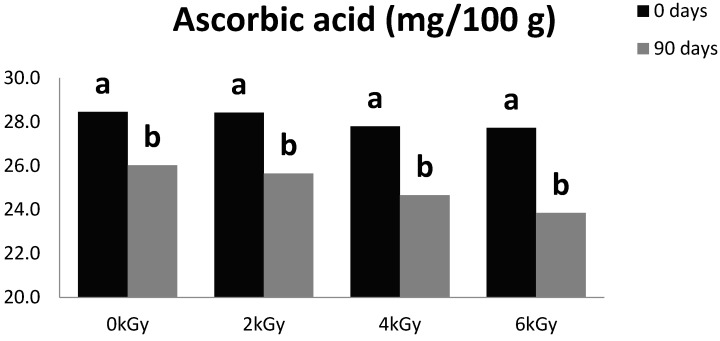
Effect of gamma radiation on ascorbic acid concentration in hot peppers during three months’ storage; **a**, **b** indicate differences between these pairs of treatment means at *p* < 0.05.

Peppers are an excellent source of ascorbic acid that besides the nutritional relevance, might contribute to prevent oxidative damage in the commodity [22]. The tested ionizing radiation doses immediately after treatments at 2-, 4- and 6 kGy did not significantly affect ascorbic acid concentrations as compared to the control. Thus, non-irradiated samples contained 28.5 ± 2.6 mg/100 g, and levels following irradiation were 28.4 ± 1.7 (2 kGy), 27.8 ± 1.7 (4 kGy), and 27.7 ± 2.0 (6 kGy). However, ascorbic acid decreased significantly (*p* < 0.05) in both control and gamma-irradiated peppers during storage for 90 days (Figure 3). The general tendency for the amount of ascorbic acid that was lost was similar for all the irradiation treatments and it was highest (14.0%) at 6 kGy. Since ascorbic acid is highly prone to oxidation upon wounding [23], the modest reduction in treated samples is an indication that gamma radiation at tested doses does not cause significant injuries in pepper fruits.

So far, research information regarding the effect of irradiation doses on ascorbic acid in dry hot peppers during storage is scarce. However, Bib et al. [24] reported that ascorbic acid concentration decreased by 7% in dried garlic powder irradiated at 1 kGy during five months’ storage. Similarly, Calucci et al. [21] found that ascorbic acid concentration decreased in different aromatic herbs and spices an average of 21% when these were irradiated at a dose of 10 kGy and stored for three months. Changes reported for total ascorbate in orange juice that was gamma irradiated up to 8.7 kGy revealed an approximate linear loss of 2.7% for each kGy increase [25].

### 3.4. Total Phenolic Compounds

**Figure 4:**
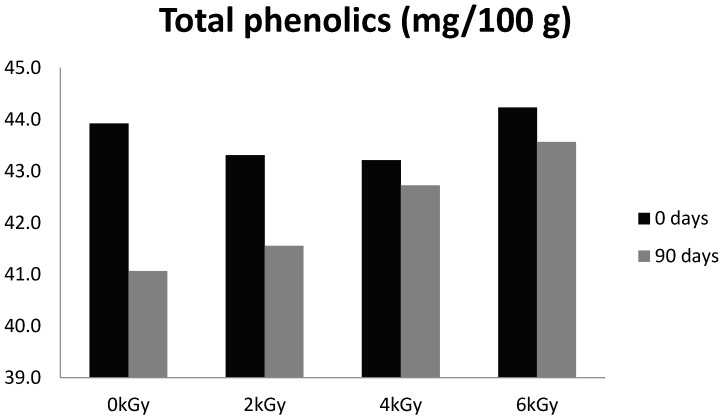
Effect of gamma radiation on total phenolic contents in hot peppers during three months’ storage.

Peppers contain phenolic compounds (mainly flavonoids), which play important roles in human health as antioxidants, and they possess anti-inflammatory, anti-allergic, anti-viral, and anti-bacterial activities [1]. The ionizing radiation treatments did not cause marked modifications in total phenolic compounds at day 0. The concentration in control non-irradiated group was 43.9 ± 12.5 mg/100 g, and attained 44.2 ± 11.9 mg/100 g after a radiation dose of 6 kGy. As shown in Figure 4, the concentration of total phenolic compounds during storage showed somewhat greater stability in irradiated samples (fell only by 1 to 4%) than in the control group (loss of 6.5%). Therefore, these results indicated that irradiation up to 6 kGy is effective for the preservation of total phenolic contents in hot peppers even though there was oxygen inside the package.

Limited information is available in the literature on the effect of gamma radiation on total phenolic contents of dry hot peppers and other spices. The results of present study are in line with the findings of Abrar et al. [26] that no significant changes in total phenolic compounds were observed in red chilies irradiated up to 6 kGy during three months’ storage. In contrast, Variyar et al. [27] found increased phenolic acid concentrations in cloves and nutmeg after irradiation. They further revealed that increase in phenolic contents was associated with the degradation of tannins in these spices. Similarly, Harrison and Were [28] also reported increase in total phenolic contents in almond skin after irradiation at 4 kGy.

## 4. Conclusions

Overall, neither decontamination treatment by gamma irradiation nor subsequent ambient storage could appreciably influence the contents of functional components in hot peppers, except for some losses of carotenoids and ascorbic acid that did not reach the 15 percent. These results revealed that gamma irradiation up to 6 kGy can maintain the quality of hot peppers to meet the export markets requirements.

## Figures and Tables

**Table 1:** Anova table for a 2-factor analysis of variance on chemical data showing *p* values.

Source	DF	Capsaicin	Dihydrocapsaicin	Carotenoids	Ascorbic Acid	Phenolics
Radiation (A)	3	0.7274	0.7588	0.7260	0.0885	0.9828
Storage (B)	1	0.5322	0.4103	0.0489 *	0.0001 *	0.6193
AB	3	0.9751	0.9766	0.9582	0.7011	0.9908

* indicates a statistically significant effect.
